# Nursing Diagnoses and Interventions in the Field of Action for Patients Undergoing Renal Replacement Therapy: A Scoping Review

**DOI:** 10.1002/nop2.70280

**Published:** 2025-07-31

**Authors:** Miguel Angel Cuevas‐Budhart, José Ramón Paniagua Sierra, Renata Cedillo‐Flores, Sonsoles Hernandez‐Iglesias, Almudena Crespo Cañizarez, Maria Alina Renghea, José Antonio Herrero Calvo, Vicente Beneit Montesinos, Mercedes Gómez Del Pulgar G

**Affiliations:** ^1^ Unidad de Investigación Médica en Enfermedades Nefrológicas, CMN S XXI Mexico; ^2^ Facultad de Estudios Superiores Iztacala, Universidad Nacional Autónoma de México; ^3^ Universidad Francisco de Vitoria Madrid Spain; ^4^ Hospital Clínico San Carlos Madrid Spain; ^5^ Facultad de Enfermería, Fisioterapia y Podología de la Universidad Complutense de Madrid Madrid Spain

**Keywords:** haemodialysis, kidney transplant, NANDA, nephrology, nursing diagnoses, nursing interventions, peritoneal dialysis

## Abstract

**Aim:**

To identify the primary nursing diagnoses and interventions related to patients with chronic kidney disease undergoing renal replacement therapy, organised according to Marjory Gordon's functional health patterns through a scoping review.

**Methods:**

A scoping review was conducted following the JBI 2020 Guidelines and the PRISMA ScR extension. Articles published between 2014 and 2021 were included from the Web of Science, Scopus and PubMed databases. The search strategy utilised DeCS and MeSH descriptors with Boolean operators AND/OR. Articles in English and Spanish authored by nurses and featuring retrospective, prospective or cross‐sectional designs were selected. After extracting the main diagnoses and interventions, a nominal group of nephrology experts discussed the findings, followed by a Delphi technique to achieve consensus.

**Results:**

A total of nine studies were included, eight from Brazil and one from Turkey, which reflects a possible geographic concentration bias. Fifty‐five nursing diagnoses and seventy‐six core interventions were identified and categorised into Marjory Gordon's 11 functional patterns, facilitating the structuring of nursing action fields in nephrology.

**Conclusions:**

This review established a set of standardised nursing interventions specific to patients with chronic kidney disease undergoing renal replacement therapy. Implications for health policies: The classification supports the organisation of nephrology units, reduces professional overload and enhances care quality and patient safety. The standardised taxonomy (NANDA, NIC, NOC) facilitates interdisciplinary communication and promotes advanced nursing practice.

**No Patient or Public Contribution:**

The study is a review of already published scientific articles and a review of experts and researchers who contributed to the document. For this reason, an express declaration of the patient, user or caregivers is not required since there was no participation in the study.

## 1 Introduction

Chronic kidney disease (CKD) has been recognised as a growing global public health issue due to its increasing incidence and prevalence. The worldwide average prevalence of patients treated with dialysis or transplants is estimated at 823 per million people, with variation across regions (Bello et al. 2024; Bradley et al. 2021; Kovesdy 2022; Méndez‐Durán et al. 2014). CKD leads to a progressive and irreversible deterioration in renal function, which, if untreated, results in death (Gansevoort et al. 2013). This condition not only affects patients and families but also imposes a significant burden on healthcare systems (Ángel et al. 2016; De Brito Poveda et al. 2014).

Nursing practice plays a critical role in the management of CKD, especially in the context of renal replacement therapy (RRT), which includes haemodialysis, peritoneal dialysis and kidney transplantation. Despite technological advances, the morbidity and mortality associated with these therapies remain high (Cuevas‐Budhart et al. 2019; Cuevas‐Budhart et al. 2022; Ku et al. 2019; Liang et al. 2011).

In response to the complexity of nephrology care, standardised nursing taxonomies such as NANDA International (NANDA‐I), Nursing Interventions Classification (NIC) and Nursing Outcomes Classification (NOC) (NANDA International 2019) have been developed to guide nursing practice and ensure continuity and quality of care (Benton et al. 2020; Forero‐Villamil et al. 2016; Sa'adeh et al. 2018). The use of such classifications allows for a common professional language to deliver care, facilitating interdisciplinary communication and documentation (Gomez del Pulgar et al. 2022; Llewellyn 2019; Sa'adeh et al. 2018).

The predialysis phase is crucial for early intervention and education. Nephrology nurses have been shown to significantly impact patient outcomes through timely assessments and individualised care planning (Forero Villalobos and Barrios Araya 2016; Polanco et al. 2021; Unsworth et al. 2022).

Therefore, the aim of this study was to identify the principal nursing diagnoses and interventions in patients with CKD undergoing RRT, organised according to Marjory Gordon's functional health patterns (Enfermeros et al. 2003), through a scoping review that integrates both scientific literature and expert consensus.

## 2 Methods

This scoping review was conducted in accordance with the JBI Manual for Evidence Synthesis (2020) and the PRISMA‐ScR, the Preferred Reporting Items for Systematic Reviews and Meta‐Analyses extension for Scoping Reviews. The methodology was rigorously structured to ensure transparency and reproducibility (Cocchiara et al. 2020; Page et al. 2021; Pham et al. 2014).

### 2.1 Eligibility Criteria

Studies eligible for inclusion were published between February 2014 and June 2021, written in English or Spanish and focused on patients diagnosed with chronic kidney disease who were undergoing renal replacement therapy, such as haemodialysis, peritoneal dialysis or kidney transplantation. Only studies with a quantitative design—either retrospective, prospective or cross‐sectional—and those authored by nursing professionals were considered. Studies that were literature reviews, opinion articles or written by individuals outside the nursing field were excluded.

### 2.2 Information Sources

The literature was collected through an extensive search across three major databases: Web of Science, Scopus and PubMed. These sources were selected for their thorough indexing of peer‐reviewed nursing and medical research relevant to the study objectives.

### 2.3 Search Strategy

The search used Medical Subject Headings (MeSH) and Health Sciences Descriptors (DeCS), along with Boolean operators (AND/OR). The keywords included nursing interventions, haemodialysis, peritoneal dialysis, kidney transplant, chronic kidney disease and renal replacement therapy (Joo and Huber 2014; Liang et al. 2011).

### 2.4 Selection of Sources of Evidence

A total of 1188 records were initially retrieved. After removing duplicates and irrelevant items, 897 articles were screened. Two independent reviewers conducted the selection process in two phases: Title/abstract screening and full‐text review. Disagreements were resolved by consensus or adjudicated by a third reviewer. Ultimately, nine studies met the inclusion criteria.

### 2.5 Data Charting Process

Relevant data were extracted by the primary reviewers using a standardised Excel template. The extraction included the following data points: Author, year, country, study design, sample size, RRT modality and key nursing diagnoses and interventions. All data were independently reviewed and cross‐validated.

### 2.6 Data Items

The key data extracted from each study included the identified nursing diagnoses categorised under the NANDA‐I taxonomy, the corresponding nursing interventions classified by the NIC and the functional health patterns based on Marjory Gordon's framework. These data items were selected to enable a comprehensive and structured synthesis of the nursing language applied to patients with chronic kidney disease receiving renal replacement therapy.

In the interventions identified in the documents, their components were reviewed: label, definition and activities. A list was drawn up with all the interventions NIC that were considered pertinent concerning the patient with renal replacement therapy. For this, the platform of good nursing practices E‐Cuidados (Plataforma E‐Cuidados 2022) was used to identify nursing diagnoses according to the criteria established by NANDA International.

### 2.7 Synthesis of Results

The gathered data were arranged according to Marjory Gordon's 11 functional health patterns. Diagnoses were categorised as either problem‐focused or risk‐based, while interventions were grouped and refined through expert consensus.

### 2.8 Expert Consultation and Delphi Technique

To validate the findings from the included studies, a Delphi technique was implemented in two rounds. A panel of 10 external experts from various Latin American countries was selected based on the following criteria: (1) current or recent clinical practice in nephrology nursing, (2) an academic or research background in standardised nursing language and (3) familiarity with NANDA‐I, NIC and NOC taxonomies (Elsevier, 2019). The group comprised a mix of clinical nurses, nursing faculty and researchers, all with at least 5 years of experience in nephrology or nursing taxonomy. After an initial review by a nominal group, the panel engaged in two rounds of anonymous consensus assessment.

Consensus was defined as agreement by at least 80% of the experts on the relevance and applicability of each nursing diagnosis and intervention identified.

Finally, the resulting data were exhaustively analysed by the principal researchers with the help of information processing using Microsoft Excel 2022 spreadsheets.

## 3 Results

Eleven documents were identified that met the selection criteria and that had an adequate methodological quality assessment; however, two of them had another type of nursing language, which is why nine final documents were included in the review (See Figure S1).

Eight documents were identified from Brazil (89%) (Alves et al. 2014; Arreguy‐Sena et al. 2018; Campos et al. 2019; Farias et al. 2015; S. A. L. Ferreira et al. 2014; da Rosendo Silva et al. 2016; Sartori et al. 2018; Spigolon et al. 2018) and only one from Turkey (11%) (Ozdemir Koken et al. 2019).

Regarding the type of methods, five of them (56%) were cross‐sectional (da Rosendo Silva et al. 2016; Farias et al. 2015; Ferreira et al. 2014; Sartori et al. 2018; Spigolon et al. 2018). One of them (11%) was about content validation by Campos et al. (2019): A retrospective study by Ozdemir Koken et al. (2019); and finally, two articles were case studies, one of them exploratory by Debone et al. (2017) in comparison with Arreguy‐Sena et al. (2018) that sought to validate forms to subsidise the systematisation of nursing care. Nursing staff (See Table 1) wrote the nine articles.

**TABLE 1:** Characteristics of the included studies.

Author (year) country	Objective	Design	Sample size	Main results
Campos et al. (2019) Brazil	Analyse the relationship between nursing diagnoses and their defining characteristics, related or risk factors in patients on peritoneal dialysis.	Content validation study	82	Four nursing diagnoses associated with the patient on peritoneal dialysis were found, belonging to the domains: of activity/rest, elimination and exchange and nutrition. The diagnoses were: Fatigue, impaired physical mobility, constipation and fluid volume excess.
Arreguy‐Sena et al. (2018) Brazil	Create and validate forms to subsidise the systematisation of nursing care with people on haemodialysis.	Case study	18	The forms captured 43 diagnoses, 26 interventions and 78 nursing results, portraying human responses in their singularities.
da Rosendo Silva et al. (2016) Brazil	To identify the predictive factors for the establishment of nursing diagnoses in patients with renal transplantation.	Transversal study	102	In kidney transplant patients, there were 16 common diagnoses: Risk of infection, impaired urinary elimination, acute pain, impaired skin integrity, nutritional imbalance: below bodily needs, sleep pattern disorder, fatigue, impaired wandering, risk of constipation, self‐care deficit: bathroom, impaired physical mobility, anxiety, fear and knowledge deficit.
Ozdemir Koken et al. (2019) Turkey	Determine the nursing diagnoses and interventions applied to kidney transplant recipients.	Retrospective and descriptive study	100	The most used nursing diagnoses in the care of kidney transplant recipients were risk of infection, knowledge deficit, risk of bleeding, acute pain and risk of falls. The most common interventions were reviewing infection‐related laboratory findings, limiting the number of visitors and reserving time for patient questions and concerns.
Ferreira et al. (2014) Brazil	To identify the nursing diagnoses applied to kidney transplant recipients in a Brazilian hospital.	Transversal study	165	Six NDs are presented in more than 50% of the sample: Risk of infection, impaired urinary elimination, ineffective protection, self‐care deficit: bathing, impaired tissue integrity and acute pain.
Farias et al. (2015) Brazil	To identify the similarities between NANDA International nursing diagnoses and Roy's adaptation problems in chronic kidney patients on haemodialysis.	Cross‐sectional and descriptive study.	178	The similarity was found between 20 nursing diagnoses and 22 adjustment problems. Roy's modes of adaptation that presented these relationships were: Physiological, self‐conception and role function.
Sartori et al. (2018) Brazil	To identify nursing diagnoses from NANDA‐I Taxonomy II in patients treated in the Haemodynamics Unit.	Cross‐sectional and descriptive study.	100	In total, 28 nursing diagnoses were identified, being considered for discussion 13 that presented a frequency greater than 50% and represented the main adaptive problems. The protection, neurological and activity and rest components were the most frequent.
Spigolon et al. (2018) Brazil	Identify the Nursing Diagnoses of carriers of Stage 5 haemodialysis chronic kidney disease.	Cross‐sectional and descriptive study.	151	Seventeen nursing diagnoses were identified, in which the risk ones stood out, present in 100% of the individuals: risks of electrolyte imbalance; ineffective renal perfusion; infection: vascular trauma and adverse response to iodine contrast medium and that of impaired urinary elimination.
Debone et al. (2017) Brazil	Identify the main nursing diagnoses (ND) in the elderly receiving haemodialysis treatment.	Exploratory research using case studies.	28	The ND was 110, with an average of 3.9 per patient. Seven different NDs were chosen, and both: Risk of infection and Fluid Volume Excess were present in all patients (100%), and the Risk of electrolyte imbalance in 26 elderly (96.8%), were considered as the principal diagnosis.

Abbreviations: NANDA: North American Nursing Diagnosis Association; ND: Nursing diagnosis; NDs: Nursing Diagnoses.

Mainly five studies (55%) performed on haemodialysis were identified by Arreguy‐Sena et al. (2018); Farias et al. (2015); Sartori et al. (2018); Spigolon et al. (2018); Debone et al. (2017). Three studies were on kidney transplantation (33%): da Rosendo Silva et al. (2016); Ozdemir Koken et al. (2019); Ferreira et al. (2014). Finally, only one of them, a study carried out on peritoneal dialysis, was found; this one was carried out by Campos et al. (2019). This encompasses all three types of renal replacement therapy. However, greater scientific production is visualised in haemodialysis therapy.

Regarding the analysis of the studies by the driving group, a list of diagnoses and interventions carried out in the field of nephrology was identified, as well as the exhaustive analysis of the Delphi group of experts; after the online seminar, those diagnoses and interventions that were not specific to patients on renal replacement therapy were excluded.

Subsequently, after expert analysis, a list of 55 primary diagnoses of the CKD patient on RRT was obtained, of which 40 were diagnoses focused on the problem (current) and 15 risk diagnoses (See Table 2). In this same sense, the nursing interventions were grouped; for this, 76 primary interventions executed in the field of action in the patient with RRT were agreed upon after analysing the documents (See Table 3).

**TABLE 2:** Nursing diagnoses in patients with CKD in RRT.

Code	Diagnosis	Code	Diagnosis	Code	Diagnosis
00137	Chronic sorrow	00013	Diarrhoea	00155	Risk for falls
00069	Ineffective coping	00214	Alteration in comfort.	00062	Risk for caregiver role strain
00053	Social isolation	00029	Decreased cardiac output	00028	Risk for deficient fluid volume
00146	Anxiety	00132	Acute pain	00025	Risk for imbalanced fluid volume
00119	Chronic low self‐esteem	00133	Chronic pain	00195	Risk for electrolyte imbalance
00120	Situational low self‐esteem	00136	Grieving	00047	Risk for impaired skin integrity
00061	Caregiver role strain	00011	Constipation	00015	Risk for constipation
00126	Deficient knowledge	00026	Excess fluid volume	00004	Risk for infection
00097	Deficit Recreation	00078	Ineffective health maintenance	00179	Risk for unstable blood glucose level
00102	Feeding self‐care deficit	00092	Activity intolerance	00203	Risk for ineffective renal perfusion.
00108	Bathing self‐care deficit	00099	Ineffective family health management	00228	Risk for ineffective peripheral tissue perfusion
00027	Deficient fluid volume	00134	Nausea	00206	Risk for bleeding
00002	Imbalanced Nutrition: Less Than Body Requirements	00032	Ineffective breathing pattern	00054	Risk for loneliness
00124	Hopelessness	00065	Ineffective sexuality pattern	00038	Risk for physical trauma
00016	Impaired urinary elimination	00204	Ineffective peripheral tissue perfusion	00213	Risk for vascular trauma
00046	Impaired skin integrity	00066	Spiritual distress		
00085	Impaired physical mobility	00148	Fear		
00033	Impaired spontaneous ventilation	00118	Disturbed body image		
00030	Impaired gas exchange	00122	Disturbed Sensory Perception^a^		
00098	Impaired home maintenance	00198	Disturbed sleep pattern		

*Note:* The code corresponds to an international and standardised five‐digit number that identifies each nursing diagnosis.

^a^ Visual, auditory, kinesthetic, gustatory, tactile, olfactory.

**TABLE 3:** Nursing Interventions in patients with CKD in RRT.

Code	Intervention	Code	Intervention	Code	Intervention
4420	Agreement with the patient	5618	Teaching procedure/treatment	1910	Management of acid–base balance
2300	Medication administration	5602	Teaching: disease process	4500	Management of constipation/faecal impaction
7610	Laboratory analysis at the patient's bedside	4920	Active listening	1260	Weight management
7040	Primary Caregiver Support	4410	Setting common goals	3550	Pruritus management
5270	Emotional Support	4235	Phlebotomy: cannulated route	1570	Management of vomiting
5250	Support in decision making	7110	Encourage family involvement	4240	Dialysis Access Maintenance
5240	Counselling	1400	Encourage body mechanics	5395	Improve self‐efficacy
5246	Nutritional advice	7320	Case management	5220	Improve body image
7380	Assistance for financial resources	6610	Risk identification	5515	Improve access to health information
1800	Help with self‐care	7330	Cultural mediation	5230	Improve coping
1801	Help with self‐care: bathing/hygiene	7690	Interpretation of laboratory data	2020	Electrolyte monitoring
4470	Self‐Modification Assistance	6482	Environmental management: comfort	4130	Liquid monitoring
5480	Values Clarification	6486	Environmental management: security	6680	Vital signs monitoring
7710	Collaboration with the doctor	2000	Electrolyte management	1160	Nutritional monitoring
8180	Phone Consultation	2120	Management of hyperglycaemia	4035	Capillary blood sample
6540	Infection control	4170	Management of hypervolemia	7370	Discharge planning
1876	Catheter care	2130	Management of hypervolemia	5400	Enhancement of self‐esteem
3440	Incision Site Care	4180	Management of hypovolemia	5100	Enhancement of socialisation
5310	Give hope	2380	Medication management	6550	Protection against infections
5820	Decreased anxiety	1100	Nutrition management	1460	Progressive muscle relaxation
4028	Decreased bleeding: wounds	7880	Technology management	8020	Multidisciplinary meeting on care
5510	Education for health	1450	Nausea management	2150	Peritoneal dialysis therapy
5614	Education: prescribed diet	4120	Liquid handling	2100	Haemodialysis therapy
5606	Individual Education	2080	Fluid/electrolyte management	3590	Skin surveillance
5616	Education: prescribed medications	7820	Sample management		
5610	Presurgical Education	1400	Pain management		

*Note:* The nursing intervention code is an international number, composed of four digits to identify each of the NICs.

With the results obtained, the interventions were associated with each of the functional patterns to order them according to the affected pattern. In this sense, 202 Nursing Interventions (NIC) have been determined and classified into Marjory Gordon's 11 functional patterns, which reflect biopsychosocial and spiritual alterations of patients with kidney disease with RRT, as can be seen in Table S1.

## 4 Discussion

This scoping review contributes to the growing body of evidence on nursing care in nephrology by identifying and organising nursing diagnoses and interventions relevant to patients with chronic kidney disease (CKD) undergoing renal replacement therapy (RRT). Utilising standardised taxonomies (NANDA‐I, NIC) and applying Marjory Gordon's functional health patterns provide a comprehensive and systematic framework for structuring nursing care.

The findings align with existing literature highlighting the complexity of care needed for CKD patients. For instance, Farias et al. (2015) and da Rosendo Silva et al. (2016) identified fluid volume excess, risk of infection and impaired mobility as recurring nursing concerns, which correspond with this study's findings. By categorising these issues under Gordon's patterns, this review promotes more organised care planning and prioritisation in clinical practice.

Furthermore, the prevalence of Brazilian studies indicates a robust research environment in that region. However, this may also restrict the generalisability of findings to other healthcare systems. Other reviews, such as Spigolon et al. (2018), have urged for greater representation of diverse cultural and clinical contexts in nephrology nursing research. The scarcity of studies on peritoneal dialysis, as also noted by De Brito Poveda et al. (2014), highlights a persistent gap in the literature that needs to be addressed.

From a clinical perspective, classifying 55 nursing diagnoses and 76 interventions enables nurses to anticipate patient needs, standardise assessments and tailor interventions to individual functional patterns. This approach supports safer, evidence‐based care and reinforces the role of nurses in managing the complexity of chronic diseases. It also lays the groundwork for developing clinical guidelines and decision‐making tools in nephrology.

The integration of expert consensus through the Delphi technique further enhances the practical relevance of the results. Expert validation ensures that the identified diagnoses and interventions are not only theoretically sound but also applicable in real‐world nephrology settings.

As highlighted in similar scoping reviews by Gerchow et al. (2020), the adoption of standardised nursing languages contributes to professional development, facilitates communication across multidisciplinary teams and enhances patient outcomes. The relevance of applying NANDA‐I taxonomy to structure nursing diagnoses has also been demostrated in intensive care contexts, supporting its validiy beyond nephrology (Ferreira et al. 2016). These findings should inform not only clinical care but also nursing education, helping future professionals internalise a taxonomy‐driven approach.

Given the underrepresentation of certain modalities and regions, future studies should aim to expand the evidence base, especially regarding peritoneal dialysis and kidney transplantation care. Multinational collaborations could provide more generalisable insights and strengthen the global applicability of nursing taxonomies in nephrology.

In summary, this review aligns with its objective of mapping the current landscape of nursing interventions and diagnoses in RRT. It provides tools that can enhance the quality and consistency of nursing care in nephrology, foster professional growth and support health policy initiatives that recognise the complexity and value of nephrology nursing.

### 4.1 Implications for Clinical Practice and Future Research

This review provides a practical foundation for enhancing clinical decision‐making and care delivery in nephrology nursing. By structuring nursing diagnoses and interventions through standardised taxonomies and Gordon's patterns, nurses are equipped with tools that promote comprehensive, person‐centred care. This classification facilitates clearer documentation, improved interdisciplinary collaboration and the development of protocols tailored to haemodialysis, peritoneal dialysis and transplant settings.

Further research is needed to validate and expand the proposed framework across diverse populations and modalities, particularly in peritoneal dialysis and posttransplant care. Broader international studies could strengthen the global relevance and utility of standardised nursing interventions in renal care.

### 4.2 Implications for Health Policies

The use of standardised languages offers an opportunity for the development and qualification of nursing knowledge within the relevant field. This classification of nursing diagnoses and interventions for the care of patients with CKD could enable dialysis and transplant units to be organised effectively, reducing work overload and stress for professionals while enhancing patient care and safety. It also facilitates disciplinary relationships, a unified record of care, monitoring and evaluation. Furthermore, having standardised care can help ensure the quality of health care and nursing practices, achieving greater access and universal health coverage. It also optimises the use of human resources with an emphasis on advanced nursing practice, simultaneously highlighting its contribution to health care.

### 4.3 Limitations

The studies included in this review were predominantly conducted in two countries, limiting the generalisability of the findings to other healthcare systems and cultural contexts. Moreover, the absence of statistical meta‐analysis restricts the ability to identify cross‐study patterns or trends in nursing diagnoses and interventions. Future research should incorporate broader geographic representation and quantitative synthesis to enhance the applicability of standardised nursing language globally.

## 5 Conclusions

This scoping review provides a clinically relevant synthesis of standardised nursing diagnoses and interventions for patients undergoing renal replacement therapy. By aligning care with functional health patterns, nurses are better prepared to address the complex needs of patients in haemodialysis, peritoneal dialysis and posttransplant settings.

The framework supports structured assessment, enhances clinical reasoning and contributes to safer, evidence‐based care. Its integration into daily practice can guide nursing protocols, improve patient outcomes and strengthen the role of nursing in multidisciplinary nephrology teams.

## Author Contributions

**Miguel Angel Cuevas‐Budhart:** study design; **Miguel Angel Cuevas‐Budhart** and **Almudena Crespo Cañizarez:** data collection; **Sonsoles Hernandez‐Iglesias, R.O.M., Maria Alina Renghea** and **José Antonio Herrero Calvo:** data analysis; **Mercedes Gómez Del Pulgar G** and **José Antonio Herrero Calvo:** study supervision; **Miguel Angel Cuevas‐Budhart** and **Renata Cedillo‐Flores:** manuscript writing; **José Ramón Paniagua Sierra** and **J.B.M.:** critical revisions for important intellectual content.

## Conflicts of Interest

The authors declare no conflicts of interest.

## Supporting information


**Table S1.** Nursing Interventions (NIC) due to altered functional patterns in patients with kidney disease with RRT.

**Figure S1.** Flow diagram.

## Data Availability

This article corresponds to a review of documents supported by scientific articles found in various databases, in addition to the consultation of two platforms for contrast and discussion with experts of said results. The data are available below:
Arreguy‐Sena et al. (2018) Construction and validation of forms: systematisation of the care of people under haemodialysis. Scielo DOI: https://doi.org/10.1590/0034‐7167‐2015‐0130
Campos et al. (2019). Pacientes em diálise peritoneal: associação entre diagnósticos de enfermagem e seus componentes. DOI: https://doi.org/10.1590/1982‐0194201900090
Debone et al. (2017). Nursing diagnosis in older adults with chronic kidney disease on haemodialysis. DOI: https://doi.org/10.1590/0034‐7167‐2017‐0117
Farias et al. (2015). Nursing diagnoses and adaptation problems among chronic renal patients. DOI: https://doi.org/10.17533/udea.iee.v33n1a14
Ferreira et al. (2014). Nursing diagnoses among kidney transplant recipients: evidence from clinical practice. *DOI*: https://doi.org/10.1111/2047‐3095.12006
Ozdemir Koken et al. (2019). Nursing Diagnoses and Interventions in Kidney Transplant Recipients: A Retrospective Study. *DOI*: https://doi.org/10.1016/J.TRANSPROCEED.2019.03.047
da Rosendo Silva et al. (2016). Predictive factors for the Nursing Diagnoses in people living with Acquired Immune Deficiency Syndrome. *DOI*: https://doi.org/10.1590/1518‐8345.1103.2712
Sartori et al. (2018). Nursing diagnoses in the hemodynamics sector: an adaptive perspective. *DOI*: https://doi.org/10.1590/S1980‐220X2017006703381
Spigolon et al. (2018). Nursing diagnoses of patients with kidney disease undergoing haemodialysis: a cross‐sectional study. *DOI*: https://doi.org/10.1590/0034‐7167‐2017‐0225
e‐ Cuidados, Plataform. (2022) Available: https://www.ieinstituto.es/areas‐de‐desarrollo/observatorio‐enfermero/prescripcion‐enfermera/plataforma‐e‐cuidados
*NNNConsult*. Elsevier. (2019). Available at https://www.nnnconsult.com/
