# Supplementary material for: Nursing Diagnoses and Interventions in the Field of Action for Patients Undergoing Renal Replacement Therapy: A Scoping Review
Source: Nurs Open. 2025 Jul 31;12(8):e70280. doi: 10.1002/nop2.70280 (PMC12313543; doi:10.1002/nop2.70280)
Supplement: Supplementary file 1 — Table S1. Nursing Interventions (NIC) due to altered functional patterns in patients with kidney disease with RRT. [file NOP2-12-e70280-s001.docx]

**Supplementary Figure S1**

**Identification of studies via databases and registers**

Records removed *before screening*:

Duplicate records removed

(n=9)

Records removed for other reasons; were not related to the discipline (nursing) (n=282)

(n = 291)

Records identified from: 1188

**Identification**

Records excluded; did not meet eligibility criteria

(n = 856)

Records screened

(n = 897)

Reports sought for retrieval

(n =41)

Reports not retrieved; were not related to chronic kidney disease

(n = 30)

**Screening**

Reports assessed for eligibility

(n =11)

Reports excluded:

Reason: Other nursing language (n = 2)

Studies included in review

(n = 9)

**Included**

*From:*  Page MJ, McKenzie JE, Bossuyt PM, Boutron I, Hoffmann TC, Mulrow CD, et al. The PRISMA 2020 statement: an updated guideline for reporting systematic reviews. BMJ 2021;372:n71. doi: 10.1136/bmj.n71
