# Supplementary material for: Nursing Diagnoses and Interventions in the Field of Action for Patients Undergoing Renal Replacement Therapy: A Scoping Review
Source: Nurs Open. 2025 Jul 31;12(8):e70280. doi: 10.1002/nop2.70280 (PMC12313543; doi:10.1002/nop2.70280)
Supplement: Supplementary file 2 — Figure S1. Flow diagram. [file NOP2-12-e70280-s002.docx]

**Table S1. Nursing Interventions (NIC) due to altered functional patterns in patients with kidney disease with RRT.**

| 1. Health perception and health management *n=39* | 1. Nutrition and metabolism *n=31* | 1. Activity and exercise *n=42* | 1. Sleep and rest *n=02* | 8. Roles and relationships *n=11* |
| --- | --- | --- | --- | --- |
| Agreement with the patient | Medication administration | Medication administration | Medication administration  Environmental Management: Comfort. | Primary Caregiver Support |
| Laboratory analysis at the patient's bedside | Laboratory analysis at the patient's bedside | Laboratory analysis at the patient's bedside |  | Support in decision-making |
| Caregiver Support | Nutritional advice | Primary Caregiver Support | 1. **Cognition and Perception *n=23*** | Counseling |
| Emotional Support | Infection control | Help with self-care | Medication administration | Phone Consultation |
| Counseling | Education: prescribed diet | Bathing self-care deficit | Emotional Support | Teaching procedure/treatment |
| Self-Modification Assistance | Phlebotomy: cannulated route | Collaboration with physician | Support in decision-making | Teaching: disease process |
| Phone Consultation | Interpretation of laboratory data | Decreased anxiety | Nutritional advice | Encourage family involvement |
| Infection control | Electrolyte management | Decreased bleeding: wounds | Phone Consultation | Case management |
| Catheter care | Hyperglycemia Management | Education: prescribed diet | Education: prescribed diet | Cultural mediation |
| Incision Site Care | Hypervolemia Management | Individual Education | Individual Education | Environmental management: safety |
| Decreased bleeding: wounds | Hypoglycemia Management | Education: prescribed medications | Education: prescribed medications | Improve access to health information |
| Education for health | Hypovolemia Management | Teaching procedure/treatment | Teaching procedure/treatment | **9.**  **Sexuality and reproduction –*n=02*** |
| Teaching procedure/treatment | Medication Management | Teaching: disease process | Teaching: disease process | Improve coping |
| Teaching: disease process | Nutrition management | Setting common goals | Case management | Enhancement of self-esteem |
| Phlebotomy: cannulated route | Nausea management | Phlebotomy: cannulated route | Environmental management: comfort | **10.**  **Coping and stress tolerance *n=18*** |
| Encourage family involvement | Fluid management | Encourage body mechanics | Environmental management: safety | Agreement with the patient |
| Case management | Fluid/electrolyte management | Interpretation of laboratory data | Hypovolemia Management | Emotional Support |
| Risk identification | Sample management | Environmental management: comfort | Hypoglycemia Management | Support in decision-making |
| Cultural mediation | Management of acid base balance | Environmental management: safety | Medication Management | Counseling |
| Interpretation of laboratory data | Pruritus management | Electrolyte management | Sample management | Assistance for financial resources |
| Environmental management: safety | Management of vomiting | Hyperglycemia Management | Management of vomiting | Decreased anxiety |
| Hyperglycemia Management | Dialysis Access Maintenance | Hypervolemia Management | Fluid monitoring | Education for health |
| Hypoglycemia Management | Improve self-efficacy | Hypoglycemia Management | Vital signs monitoring | Pre-surgical Education |
| Medication management | Electrolyte monitoring | Hypovolemia Management | Discharge planning | Teaching procedure/treatment |
| Fluid management | Fluid monitoring | Medication Management | Protection against infections | Teaching: disease process |
| Fluid/electrolyte management | Vital signs monitoring | Nutrition management | Multidisciplinary meeting on care | Active listening |
| Management of acid base balance | Nutritional monitoring | Technology management | 1. **Self-perception and self-concept *n=14*** | Encourage family involvement |
| Pruritus management | Capillary blood sample | Nausea management | Emotional Support | Case management |
| Dialysis Access Maintenance | Protection against infections | Fluid management | Support in decision-making | Cultural mediation |
| Improve access to health information | Peritoneal dialysis therapy | Fluid/electrolyte management | Counseling | Improve body image |
| Electrolyte monitoring | Hemodialysis therapy | Sample management | Values Clarification | Improve access to health information |
| Fluid monitoring | 1. **Elimination *n=12*** | Pain management | Give hope | Improve coping |
| Vital signs monitoring | Medication administration | Management of acid base balance | Pre-surgical Education | Vital signs monitoring |
| Nutritional monitoring | Infection control | Weight management | Teaching procedure/treatment | **11.**  **Values and belief *n=08*** |
| Capillary blood sample | Electrolyte management | Management of vomiting | Teaching: disease process | Emotional Support |
| Enhancement of socialization | Medication Management | Electrolyte monitoring | Environmental management: safety | Support in decision-making |
| Protection against infections | Fluid management | Fluid monitoring | Improve coping | Give hope |
| Peritoneal dialysis therapy | Fluid/electrolyte management | Vital signs monitoring | Vital signs monitoring | Active listening |
| Hemodialysis therapy | Management of constipation/fecal impaction | Nutritional monitoring | Nutritional monitoring | Environmental management: comfort |
|  | Fluid monitoring | Capillary blood sample | Enhancement of self-esteem | Environmental management: safety |
|  | Nutritional monitoring | Peritoneal dialysis therapy | Progressive muscle relaxation | Management of constipation/fecal impaction |
|  | Protection against infections | Hemodialysis therapy |  | Improve coping |
|  | Hemodialysis therapy |  |  |  |
|  | Surveillance: skin |  |  |  |
